# 

**Published:** 1968-04

**Authors:** 


### *Contents*

| | | Page |
|---|---|---|
| A COMPARATIVE MORTALITY WITH SPECIAL REFERENCE TO BRISTOL | *R. C. Wofinden* | 1 |
| STARTING AFRESH : AN UNFINISHED HISTORY | *G. P. Tonkin* | 12 |
| REVIEW — A FORTUNATE MAN | *Berger* | 15 |
| OBITUARY | | 16 |
| CORRESPONDENCE | | 20 |
| NOTES AND NEWS | | 21 |

